# Disaster and Emergency Medicine – A Conceptual Introduction

**DOI:** 10.3389/fpubh.2013.00044

**Published:** 2013-11-05

**Authors:** Kobi Peleg

**Affiliations:** ^1^Disaster Medicine Department, School of Public Health, Faculty of Medicine, Tel-Aviv University, Tel-Aviv, Israel; ^2^National Center for Trauma and Emergency Medicine Research, The Gertner Institute for Health Policy and Epidemiology, Tel Hashomer, Israel

**Keywords:** disasters, disaster medicine, mass casualty incidents, emergency medicine, emergency medical services

Disasters and Mass Casualty Incidents (MCI) occur out of the blue, most times without warning, and in a wide scope of locations. Each disaster is different from the rest, both in the past and in the future. This creates a situation where the experience accumulated in one place is relatively small and localized in the long-haul, and it is therefore our duty to learn and share information, knowledge and experience accumulated in the over time, from all disasters and events, with one major common shared goal in mind – to allow everyone to be better prepared for the future and allow for a greater ability to save lives and prevent injuries and damage.

According to the ReliefWeb, a service conducted by OCHA (Office for the Coordination of Humanitarian Affairs), in the years 2002–2011 the annual average number of people who were killed in natural disasters was 107,000 worldwide. During this period of time 268 million people became victims of natural disasters worldwide. The economic damages because of natural disasters were estimated to be an annual average of 143 billion USD (1).

In recent years, because of media development, the advance in communication and the rise in world population density, among other reasons, the issue of disasters as well as disaster medicine have been brought to the center of public awareness. The practice of disaster and emergency management, as well as that of disaster medicine has gained knowledge and momentum which lead to an improvement of capabilities in recent decades.

Disaster medicine and MCI came to be a part of modern medicine which has seen some great advances. With those, unfortunately, there came a tidal wave (sometimes literally) of challenges. The number of natural disasters wreaking havoc on the Earth’s populations has dramatically risen over the past 50 years (2). Our global infrastructure has become more inter-linked and as a result more vulnerable to a wide variety of threats, both natural and man-made.

Disasters have several phases, each requiring different actions and approaches. Disaster preparedness is the phase focusing on prevention (when possible), planning, education, training, etc., of disasters in the aim of preventing, or at least mitigating injuries, loss of lives and damage from disasters, as much as possible. Disaster preparation centers on implementing the plan devised at the planning stage, including drills and exercises. Disaster response is the immediate action taken following a disaster. The focus is saving lives. Disaster recovery is the effort to return to normal life after the disaster. It begins with early recovery, which is parallel to the response phase, and continues with the long and resource-consuming recovery phase, designed to restore peoples’ lives back to normal (3). To that effect, many components need attention, from shelters, infrastructure, work places, healthcare, water supply, energy resources, and many more. Recovery is completed by taking actions to prevent a recurrence of the disaster in the future, or to mitigate the impacts of future recurring incidents.

At the basis of dealing with these complex challenges, from the aspect of saving human lives and mitigating the enormous costs of disasters and emergencies, lies the field known as Disaster and Emergency Medicine. This field of specialty has seen many changes from the days of the Napoleonic wars, when soldiers where for the first time treated on the battlefield, and the concept of triage was created. The emphasis of disaster medicine is on providing medical attention to survivors, but also on planning the medical response to future disasters, preparing for them, dealing with them and handling their recovery. When evacuation is needed, disaster medicine specialists become an integral part of the emergency management system, providing vital information and assistance.

Emergency Medicine is the field of practice concerned with the assessment, stabilization, diagnosis, and disposition of people with acute illness and injury (4). Studies have shown that time is a leading factor in determining the future potential outcome of medical treatment during emergencies, a point which needs to be central to all personnel – both medical and non-medical.

Thus dealing with disasters and emergencies is basically the combination of treating both the individual patient quickly, but at the same time ensuring the entire wounded population is being taken into consideration. This inherent conflict of dealing with both an individual patient as well as an increase of normal hospital and emergency activity is a great challenge, which requires planning and training, to allow for adequate resources for this surge.

The general influx of injured, their families, media, all entering the hospital at the same time can cause significant disturbances to the facility’s function. The ability to effectively handle such an unplanned and unexpected event is known as surge capacity (5). Medical and Mental-health triage are fundamental factors in establishing surge capacity.

Disaster and Emergency Medicine plays a part in all the stages of dealing with an event – from the early stages of planning and policy-making toward potential prevention, going through preparedness and culminating with response when medical personnel are heavily involved in life-saving activities. Recovery efforts and getting life back to normal are connected to medical professionals as well, with an emphasis on cooperation with mental-health professionals.

Dealing with disaster is essentially dealing with highly complex and unexpected events. This requires cooperation of many professionals. When a disaster takes place at a certain location, the medical needs of the population affected change. As a result a medical contingency plan is needed to allow for those needs to be met, at least partially. This can only be done if medical professionals are in open and clear communications with other experts from the field of emergency management, government personnel, law enforcement, armed forces, etc. Lack of compunctions at the planning and execution stages can dramatically harm the population affected by disasters.

Disasters have far reaching effects for the population. Psychological and behavioral support must be part of the medical support given to survivors en route to recovery. Mental-health implications are a serious part of the post-event causes of concern, especially in terms of insuring a successful recovery. High percentages of injuries in MCIs are people with Post Traumatic Disorders or Acute Stress Reaction (6).

Resilience plays a major part in the community affected by disaster. Resilience has many faces: a social aspect, an economic aspect, a leadership aspect, as well as security aspects and many more. Resilience begins at the personal level, continues at the family unit, and culminates at the community level and then spreads to the wider community level and vise versa. In recent years, resilience is a major issue in disasters (7).

All the complexities regarding the treatment of many injured patients at once can cause adverse affects to those treating them. The care for first responders and medical personnel is a field of growing importance, especially as events become more frequent and complex.

As medicine focuses on caring for the individual patient, the medical personnel involved in planning for disasters must also prepare for handling the population as a whole. This challenge requires inter-disciplinary cooperation, building upon experience and a fundamental understanding of the unique characteristics of disasters as medical mass casualty events with long-term effects, both physical and mental.
